# Disorders of mitochondrial long-chain fatty acid oxidation and the carnitine shuttle

**DOI:** 10.1007/s11154-018-9448-1

**Published:** 2018-06-20

**Authors:** Suzan J. G. Knottnerus, Jeannette C. Bleeker, Rob C. I. Wüst, Sacha Ferdinandusse, Lodewijk IJlst, Frits A. Wijburg, Ronald J. A. Wanders, Gepke Visser, Riekelt H. Houtkooper

**Affiliations:** 1 Dutch Fatty Acid Oxidation Expertise Center, Department of Metabolic Diseases, Wilhelmina Children’s Hospital, University Medical Center Utrecht, Lundlaan 6, 3584 EA Utrecht, The Netherlands; 2 Dutch Fatty Acid Oxidation Expertise Center, Laboratory Genetic Metabolic Diseases, Departments of Clinical Chemistry and Pediatrics, Emma Children’s Hospital, Academic Medical Center, Meibergdreef 9, 1105 AZ Amsterdam, The Netherlands

**Keywords:** Mitochondrial long-chain fatty acid oxidation, ß-oxidation, Carnitine transport, Inborn errors of metabolism

## Abstract

Mitochondrial fatty acid oxidation is an essential pathway for energy production, especially during prolonged fasting and sub-maximal exercise. Long-chain fatty acids are the most abundant fatty acids in the human diet and in body stores, and more than 15 enzymes are involved in long-chain fatty acid oxidation. Pathogenic mutations in genes encoding these enzymes result in a long-chain fatty acid oxidation disorder in which the energy homeostasis is compromised and long-chain acylcarnitines accumulate. Symptoms arise or exacerbate during catabolic situations, such as fasting, illness and (endurance) exercise. The clinical spectrum is very heterogeneous, ranging from hypoketotic hypoglycemia, liver dysfunction, rhabdomyolysis, cardiomyopathy and early demise. With the introduction of several of the long-chain fatty acid oxidation disorders (lcFAOD) in newborn screening panels, also asymptomatic individuals with a lcFAOD are identified. However, despite early diagnosis and dietary therapy, a significant number of patients still develop symptoms emphasizing the need for individualized treatment strategies. This review aims to function as a comprehensive reference for clinical and laboratory findings for clinicians who are confronted with pediatric and adult patients with a possible diagnosis of a lcFAOD.

## Introduction

Hypoglycemia is a common, and potentially dangerous, sign in medical practice, with an extensive number of etiologies, especially in the neonatal period. When hypoglycemia is accompanied by a low concentration, or even absence, of ketone bodies in plasma and urine, hyperinsulinism and disorders of carnitine transport and mitochondrial fatty acid oxidation, including long-chain fatty acid oxidation disorders (lcFAODs), may be involved as cause. Plasma levels of free fatty acids (FFAs) are low in hyperinsulinism due to a suppression of lipolysis, while FFAs are generally high during hypoglycemia caused by lcFAODs. In lcFAODs, FFAs are mobilized during fasting but cannot be oxidized due to an autosomal recessive inherited deficiency of one of the mitochondrial ß-oxidation enzymes. Hypoglycemia, however, is only one of the signs in lcFAODs. Before lcFAODs were included in newborn screening (NBS) panels, a considerable number of patients was diagnosed in adulthood and these patients typically presented with different clinical presentations compared to patients who presented during childhood. Following the inclusion of lcFAODs in NBS panels in many countries worldwide, the number of patients diagnosed has significantly increased and the clinical spectrum has expanded, necessitating new algorithms for choosing the optimal therapeutic strategy. This review aims to serve as a comprehensive reference for clinical and laboratory findings for those who are confronted with pediatric and adult patients with a possible diagnosis of a mitochondrial lcFAOD.

## Mitochondrial long-chain fatty acid oxidation

**Fig. 1:**
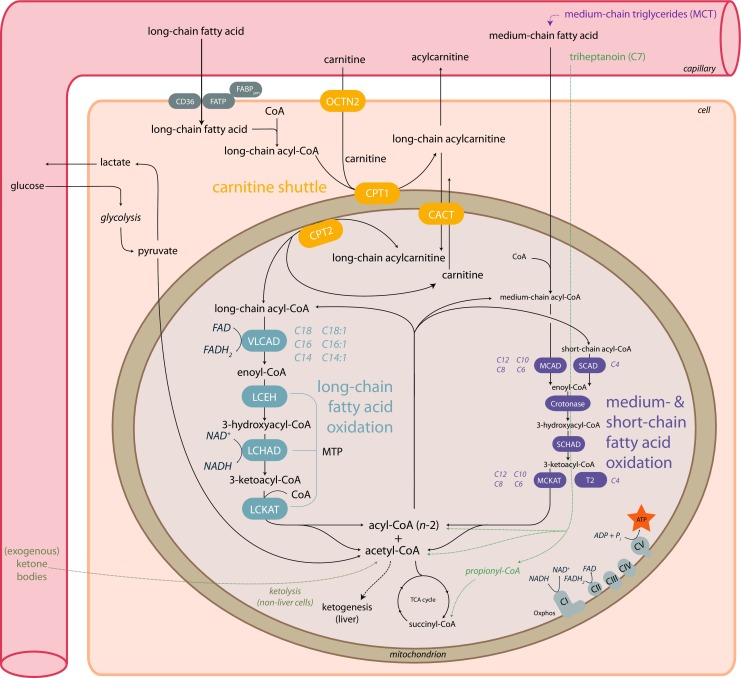
**Schematic representation of mitochondrial fatty acid oxidation in humans.** Long-chain fatty acids enter the cell from the bloodstream and enter the mitochondria through the carnitine shuttle, followed by a step-wise degradation involving a series of enzymes of the long-chain fatty acid oxidation machinery resulting in the production of acetyl-CoA. Potential treatments to produce acetyl-CoA independent of the fatty acid oxidation enzymes are indicated. These include medium-chain triglycerides, ketone bodies and triheptanoin. Abbreviations: CI-V, Complex I-V; CACT, Carnitine Acylcarnitine Translocase; CD36, Cluster of Differentiation 36; CoA, Coenzyme A; CPT1, Carnitine Palmitoyl Transferase type 1; CPT2, Carnitine Palmitoyl Transferase type 2; FABPpm, plasma membrane-associated Fatty Acid Binding Protein; FATP, Fatty Acid Transport Protein; LCEH, Long-Chain Enoyl-CoA Hydratase; LCHAD, Long-Chain 3-Hydroxyacyl-CoA-Dehydrogenase; LCKAT, Long-Chain Ketoacyl-CoA Thiolase; MCAD, Medium-Chain Acyl-CoA Dehydrogenase; MCKAT, Medium-Chain 3-Ketoacyl-CoA Thiolase; OCTN2, Organic Cation Transporter 2; SCAD, Short-Chain Acyl-CoA Dehydrogenase; SCHAD, Short-Chain 3-hydroxyacyl-CoA Dehydrogenase; T2, acetoacetyl-CoA thiolase

Mitochondrial fatty acid oxidation is an important pathway for maintaining energy homeostasis. Especially during fasting, when glucose and glycogen stores are low, fatty acid oxidation (FAO) is a significant source of energy provision in the form of adenosine triphosphate (ATP). The majority of fatty acids are stored in the body, in particular in adipose tissue, as long-chain triglycerides (LCT). As blood glucose levels drop, triglycerides in adipose tissue are hydrolyzed and free fatty acids (FFAs) are mobilized in the blood stream and become available for other tissues [1, 2]. The FFAs enter the cell via fatty acid transport systems (FAT/CD36) (Fig. 1). Before degradation or elongation can take place, FFAs need to be activated to acyl-coenzyme A (-CoA) esters by one of a variety of different chain-length-specific synthetases localized in distinct subcellular compartments [3]. Medium-chain fatty acids can freely diffuse into mitochondria, whereas long-chain acyl-CoAs cannot. Instead, long-chain fatty acids are esterified with carnitine in the cytoplasm and then are transported by the carnitine shuttle. In this shuttle, the CoA group is first exchanged for carnitine in a reaction catalyzed by carnitine palmitoyltransferase 1 (CPT1), forming acylcarnitine. CPT1 is an important regulator of FAO as it is sensitive for inhibition by malonyl-CoA, formed by carboxylation of the product of fatty acid oxidation, acetyl-CoA. The acylcarnitines are imported into the mitochondrion by carnitine acylcarnitine translocase (CACT) in exchange for a free carnitine molecule. Next, the intramitochondrial acylcarnitines are reconverted to acyl-CoAs by carnitine palmitoyltransferase 2 (CPT2). The mitochondrial acyl-CoAs can subsequently be used for energy production that occurs via degradation by a series of enzymes with different chain-length specificity. The first step concerns the dehydrogenation of acyl-CoAs by different acyl-CoA dehydrogenases. In humans, the key enzymes catalyzing this step for the different chain lengths of fatty acids are very long-chain acyl-CoA dehydrogenase (VLCAD), medium-chain acyl-CoA dehydrogenase (MCAD) and short-chain acyl-CoA dehydrogenase (SCAD) [4]. For long-chain acyl-CoAs (chain length C14-C18), VLCAD is the main enzyme [5]. In rodents, the enzyme long-chain acyl-CoA dehydrogenase (LCAD) can take over part of the VLCAD function, but in humans LCAD makes little contribution to lcFAO [6]. Indeed, nine patients that were originally reported as LCAD-deficient, diagnosed by a reduction in palmitoyl-CoA degradation [7], were reported to have normal LCAD protein levels on western blot [8] and upon subsequent analyses many were in fact VLCAD-deficient [9]. Following the initial dehydrogenation, the resulting long-chain enoyl-CoAs undergo hydration, a second dehydrogenation step, and finally thiolytic cleavage catalyzed by mitochondrial trifunctional protein (MTP) [10]. For degradation of fatty acids with shorter chain length mitochondria have different enzymes which are outside the scope of this review but are described elsewhere [11]. The MTP enzyme complex harbors activity for (a) long-chain enoyl-CoA hydratase (LCEH), (b) long-chain-(S)-3-hydroxyacyl-CoA-dehydrogenase (LCHAD), and (c) long-chain-3-ketoacyl-CoA thiolase (LCKAT). These concerted activities lead to the formation of a molecule of acetyl-CoA and a shortened acyl-CoA with two carbons less. The acetyl-CoA can enter the tricarboxylic acid (TCA) cycle for production of reducing equivalents for oxidative phosphorylation resulting in ATP production, or may enter the ketone body synthesis pathway in the liver. The heart mostly depends on FAO (60–90%), even when glucose levels are high [12]. In contrast, the brain mostly relies on the oxidation of glucose and ketone bodies. However, there is evidence of FAO in astrocytes derived from rat brain and expression of FAO enzymes in other parts of the rat brain as well as in neural cells from human fetuses [13–15].

## Clinical signs and symptoms

Inherited disorders for many of the enzymes of the FAO cascade and carnitine shuttle have been described in the past decades [16–19]. These disorders give rise to a variety of clinical features especially in organs that rely on energy production by FAO, such as the heart, skeletal muscle and liver. Signs and symptoms can manifest as early as a few hours after birth, but may also only appear during adulthood, with a variation in clinical severity between patients even within the same family [20–26]. Patients with severe phenotypes can present in infancy with hypoglycemia and lactic acidosis. In rare cases, patients also have low levels of ketones, which might therefore be misinterpreted as respiratory-chain defects [27].

The clinical presentation of lcFAODs is aspecific, but a characteristic feature of the signs and symptoms in lcFAODs is the fact that they can be provoked, or aggravated, by energy requiring states such as fasting, prolonged exercise, illness, fever or a combination of these factors. The most common clinical presentations in lcFAODs are hypoketotic hypoglycemia, cardiomyopathy and myopathy. These presentations occur in combination but also isolated:

- *Hypoketotic hypoglycemia*, sometimes resulting in convulsions, coma and brain damage, is most often seen in infancy and the early years of childhood. Hypoketotic hypoglycemia is often observed in combination with hepatomegaly, elevated transaminases, and hyperammonemia. Hepatic dysfunction is described in patients during episodes of metabolic derangement [28] and can be fatal. Chronic liver dysfunction is uncommon [23–25].
- *Cardiomyopathy*, is most often seen in the neonatal period or early childhood, but can also develop under catabolic conditions like fasting or illness later in life. The outcome can be fatal, especially in infancy. Hypertrophy of the left ventricular wall can be observed initially, which can progress to dilated cardiomyopathy in advanced disease with reduced ejection fraction. Sometimes cardiomyopathy is accompanied by pericardial effusion. Remarkably, the cardiomyopathy can be completely reversed after treatment with medium-chain triglycerides (MCT) or the odd-chain triglyceride triheptanoin [29, 30]. Rhythm disturbances have also been reported in all lcFAODs except CPT1A deficiency, and can occur with, but also without, the presence of cardiomyopathy [20, 31].
- *Myopathy*, by which we refer to any muscular sign or symptom, are common in lcFAOD patients. Patients may present with fatigue as myalgia, muscle weakness, exercise intolerance, myoglobinuria and recurrent rhabdomyolysis. Myopathy typically starts in puberty or adulthood, but can be observed at a younger age. Often myopathy is provoked by endurance-type exercise [23–25] but can also be seen after anesthesia or viral illness.

Distinctive features of the separate lcFAOD are discussed in Section 5.

## Diagnostic approach for long-chain fatty acid oxidation disorders

### Laboratory testing

When a patient is suspected of having an lcFAOD based on clinical signs and symptoms, the first laboratory test of choice is measurement of acylcarnitine levels in blood. It is assumed that the level of acylcarnitines in blood mirrors the accumulating acyl-CoA species inside mitochondria which cause the carnitine shuttle to operate in the reverse fashion [32]. CPT2 converts accumulating intramitochondrial acyl-CoAs to acylcarnitines. These are then transported back to the cytoplasm via CACT and out of the cell into the blood (Fig. 1) [33]. With the development of highly sensitive techniques based on mass spectrometry to determine acylcarnitine profiles in blood, urine or dried blood spots (DBS), a fast screening method for lcFAOD has become available [34, 35]. Abnormalities in the acylcarnitine profile are more pronounced during times of metabolic derangement but the acylcarnitine profile can be normal in patients when they are well [36–39]. This complicates the diagnosis, since false negative results can occur [40]. In addition to analysis in blood and urine, acylcarnitine profiling can also be done in cultured fibroblasts after a fatty acid loading test [41]. Most of the lcFAODs result in a distinctive acylcarnitine profile depending on which of the lcFAO enzymes is deficient (Table 1). Overall lcFAO activity can also be measured in fibroblasts; a whole cell assay, using radiolabeled fatty acids as a substrate [48, 49]. In addition, the activity of specific enzymes can be measured in lymphocytes or in skin fibroblasts, as these cell types express all lcFAO enzymes [5]. After confirmation of the enzyme deficiency, mutation analysis can uncover the underlying molecular defect in the gene coding for the mitochondrial lcFAO protein [50]. With the rapid development of quicker and low-cost sequencing tools, it is tempting to directly proceed to gene panel analysis or targeted sequencing after finding an abnormal acylcarnitine profile. Especially since the enzymatic assays are costly, laborious and only performed in a limited number of centers. It is likely that indeed genetic testing will become the first step in the diagnostic process. However, regularly new mutations and variants of unknown significance (VUS) are identified, requiring functional studies such as enzyme activity measurements or cellular flux analysis for confirmation of the diagnosis and characterization [51]. In addition, prognostic markers are still needed to predict outcome and define personalized treatment strategies [52].

**Table 1:** Acylcarnitine characteristics for lcFAODs

Deficient enzyme	Acylcarnitine profile changes	Primary marker (NBS)	References
OCTN2	C0↓	C0↓	[42]
CPT1A	C0↑,C2↓, acylcarnitine↓	C0/(C16 + C18)↑	[43, 44]
CACT	C16↑, C18↑, C18:1↑, C18:2↑	(C16 + C18:1)/C2↑	[41]
CPT2	C16↑, C18↑, C18:1↑, C18:2↑	(C16 + C18:1)/C2↑	[45]
VLCAD	C12↑, C14↑, C14:1↑, C16↑, C18↑	C14:1/C2↑	[43, 46]
MTP/LCHAD	C18OH↑, C16OH↑, C16↑, C14OH↑	C16OH↑, C18OH↑	[47]

### Newborn screening

LcFAODs are included in NBS panels in many countries, enabling prompt treatment and precautions to avoid catabolism [53, 54]. As described above, all lcFAODs can be identified by specific acylcarnitine profiles in DBS [43, 55] (Table 1). The use of DBS for screening has several advantages over screening in plasma: only a minor amount of blood is required, and it can be easily collected, shipped and stored. Cut-off values for acylcarnitines and free carnitine in DBS vary internationally; reference ranges are collected in the Region 4 Stork (R4S) project [43]. Although DBS is good matrix for detecting most lcFAODs, acylcarnitine levels in CPT2-deficient patients can be in the normal range [56]. The use of ratios of acylcarnitines improves sensitivity [46, 57]. For example for VLCAD deficiency, the ratio of tetradecenoylcarnitine (C14:1) over acetylcarnitine (C2) resulted in less false-negative results when compared to C14:1 as the only marker [46, 57]. A potential disadvantage of a more sensitive screening is that it will likely also result in identification of asymptomatic individuals who may never develop clinical symptoms. The benefit of screening for these patients is not yet clear since their risk of developing symptoms in the future is yet unknown [55, 58]. Since the introduction of NBS, several studies reported follow-up data of VLCAD-deficient patients diagnosed by NBS who were asymptomatic at time of screening and remained so for the follow-up period of the study [21, 59–61]. However, despite NBS and subsequent early diagnosis and treatment, some patients still develop severe symptoms, including fatal metabolic derangements [21, 62, 63].

## Overview of different long-chain fatty acid oxidation disorders

### Disorders of carnitine transport

#### Primary carnitine deficiency / organic cation transporter 2 (OCTN2) deficiency / carnitine transporter disorder (CTD)

##### Clinical presentation

Patients with systemic primary carnitine deficiency (also known as OCTN2 deficiency or CTD; OMIM 212140), lose carnitine in urine and as a consequence have low carnitine levels in plasma. Patients can present with hepatic, myopathic and cardiac signs and symptoms as described above (Section 3). Cardiomyopathy and cardiac arrhythmias leading to sudden death are most frequently reported [64]. Some signs and symptoms are only described in a few case reports, and include peripheral neuropathy involving both upper and lower limbs, mild developmental delay, anemia, respiratory distress and proximal muscle weakness [65–67]. The disease is particularly prevalent in the Faroe Islands, an archipelago between Scotland and Iceland with little genetic variation. A large population screening revealed a prevalence of 1:297 [68]. After a series of sudden deaths in young Faroese inhabitants who were all homozygous for the c.95A > G (p.N32S) mutation, an association was made between the use of antibiotics containing pivalic acid and development of encephalopathy and sudden cardiac arrest [69]. The following incidences of primary carnitine deficiency in newborns have been reported: 1:40,000 in Japan [70], 1:37,000–1:100,000 in Australia [71], 1:105,000 in Portugal [72], 1:142,000 in the USA [73], 1:250,000 in Germany [74] and 1:297 in the Faroe islands [68].

##### Acylcarnitine profile

In primary carnitine deficiency the transport of carnitine over the plasma membrane is impaired resulting in low intracellular carnitine concentrations. Carnitine in the blood is wasted via the urine so plasma levels of both free and acylated carnitine are extremely low (free carnitine <5 μM (reference 25–41, 43, 48–55 μM)) and carnitine levels in urine are high [64]. Other plasma acylcarnitines are also low due to impaired formation, but low levels of free carnitine (C0) remains the primary marker for diagnosis and NBS (Table 1) [42]. Transplacental transport of free carnitine from mother to infant complicates the diagnosis of patients by NBS because plasma levels can be higher resulting in false negative screening results. In addition, low levels of C0 found in NBS can also be the result of primary or secondary carnitine deficiency in the mother [75].

##### Enzymatic activity

Confirmation of primary carnitine deficiency is achieved by measuring carnitine transport in cultured fibroblasts. The severity of clinical symptoms is correlated with the level of residual transport of carnitine [76].

##### Molecular analysis

The disease is caused by mutations in the *SLC22A5* gene (OMIM 603377). Heterogeneity of mutations complicates the formation of a genotype-phenotype correlation, although it appears that the frequency of loss-of-function mutations is higher in patients who present symptomatically compared to asymptomatic women [76].

#### Carnitine Palmitoyl transferase type 1A (CPT1A) deficiency

##### Clinical presentation

Patients with CPT1A deficiency (OMIM 255120) present typically with fasting-induced hypoketotic hypoglycemia and encephalopathy without muscular involvement at an early age [77, 78]. A few cases of hepatic encephalopathy without hypoglycemia and renal tubular acidosis have also been reported [79–81]. During or after metabolic crisis, elevation of dodecanedioic acid has been observed in urine of patients [82]. Heterozygous female carriers may develop fatty liver of pregnancy when carrying a fetus with this disorder [83]. CPT1A deficiency is particularly prevalent in native populations of Alaska, Canada, Greenland and North-East Siberia [84–86]. The common c.1436C > T (p.P479L) variant in these populations causes a partially reduced CPT1A activity and is associated with infant mortality [87–90]. This mutation also results in a CPT1A protein that is less sensitive for the inhibitory effect of malonyl-CoA on CPT1A under fed conditions, resulting in a 3–4 times higher activity of CPT1A compared to wild type [91–93]. Prevalence of CPT1A deficiency in Alaskan infants is 1:780 live births [94], but is very rare among non-Inuit, non-Yupik or non-Hutterite populations. In Taiwan a prevalence of 1:769.230 is reported [95], and combined results of NBS programs in Australia, Germany and USA show an incidence of 1:750,000–1:2000,000 [55]. The prevalence in the Region 4 Stork collaborative, now called Collaborative Laboratory Integrated Reports (CLIR; www.clir-r4s.org), is 1:500,000 to 1:1,000,000.

##### Acylcarnitine profile

In CPT1A deficiency the conversion of long-chain acyl-CoAs to their corresponding acylcarnitines is impaired, resulting in low long-chain acylcarnitine levels in plasma and DBS. The ratio of C0 over long-chain acylcarnitines in DBS appears to be a good marker to screen for CPT1A deficiency (Table 1) [44, 96]. In addition, free carnitine levels in plasma are usually high [97], but if a child is in a well-fed state false negative results can still occur [37].

##### Enzymatic testing

Diagnosis should be confirmed by enzymatic and/or molecular testing. To measure CPT1A activity fibroblasts using [U-^13^C]-palmitoyl-CoA as substrate, and the amount of synthesized labeled palmitoyl-carnitine is measured by mass spectrometry [98]. Most mutations are associated with residual enzyme activity between 0 and 10% of control values.

##### Molecular analysis

All currently known CPT1-deficient patients have mutations in the *CPT1A* gene (OMIM 600528). Since the first report of a molecular defect of human CPT1A [99], many different mutations in the *CPT1A* isoform have been described [92, 100]. Disorders of CPT1B, the muscle isoform, and CPT1C, the neuronal isoform, have not been reported in humans yet. Mutations in the *CPT1A* gene are usually highly heterogeneous except for the pathogenic variant c.1436C > T (p.P479L) which is observed to have a high prevalence in the Inuit population [101] and the c. 2129G > A (p.G710E) in the North American Hutterites [85].

#### Carnitine-acylcarnitine translocase (CACT) deficiency

##### Clinical presentation

Patients with CACT deficiency (OMIM 212138) usually present in the neonatal period with severe hyperammonemia, liver failure, cardiomyopathy and respiratory distress, often with fatal outcome [23, 24, 102, 103]. Less than 60 patients have been reported in literature of whom two were detected by NBS [95, 104].

##### Acylcarnitine profile

CACT-deficient patients show elevations of C16, C18 and C18:1 carnitines. This profile, however, is not specific for CACT deficiency since CPT2 deficiency has the same profile [41].

##### Enzyme activity

CACT enzyme activity can be determined in digitonin-permeabilized fibroblasts measuring formation of radiolabeled CO_2_ from radiolabeled acetylcarnitine as a substrate [105]. There is a correlation between residual capacity of overall lcFAO and milder phenotypes [102, 105].

##### Molecular analysis

Several, mostly private mutations in the *SLC25A20* gene (OMIM 613698) have been identified in patients and most of them express severe phenotypes, which makes genotype-phenotype correlations difficult. The most frequently reported mutations include the splicing mutation c.199-10 T > G that is found in patients from East Asia (Japan, China, Vietnam) and the missense mutation c.713A > G (p.Gln238Arg) in patients of Arabic descent [102].

#### Carnitine Palmitoyl transferase type 2 (CPT2) deficiency

##### Clinical presentation

Patients with CPT2 deficiency can present with hypoglycemia, cardiomyopathy and muscular signs and symptoms [23, 24, 106]. Similar to other lcFAODs, muscular symptoms are more prominent in adulthood or adolescence and liver-related signs and symptoms are generally seen in early childhood, but patients can present with both in different stages of life. Yet, CPT2 deficiency is often referred to as either the adult/benign form or the neonatal form, suggesting these are separate identities. In contrast to other lcFAODs, congenital malformations, such as dysmorphic features, renal cysts, intracerebral and intrahepatic calcifications have been reported in several cases that suffered from the lethal neonatal form [107–110]. However, the majority of patients in literature presents in adolescence or adulthood with myopathy as first complaint [106]. Combined results of NBS programs in Australia, Germany and USA report an incidence of 1:750,000–1:2,000,000 [55], but numbers on prevalence are scarce since CPT2 deficiency has not been introduced in most NBS panels. However, many families have been reported in literature and over 300 patients have been described.

##### Acylcarnitine profile

CPT2 activity completes the carnitine cycle, by reconverting the acylcarnitines to acyl-CoA esters in mitochondria. The acylcarnitine profile of CPT2-deficient patients is identical to that of CACT patients, i.e. accumulation of C16-, C18- and C18:1-carnitine.

##### Enzymatic activity

The reaction catalyzed by CPT2 is reversible and can be measured in the forward or backward direction. In the radioisotope exchange assay, the incorporation of labeled carnitine in palmitoyl-carnitine is reflecting (backward) CPT2 activity [111, 112]. Methods to measure the physiological forward reaction have also been developed. Fibroblasts or lymphocytes are incubated with palmitoyl-carnitine, and formed palmitoyl-CoA is measured using (ultra)high performance liquid chromatography ((U)HPLC) in a medium where CPT1 is blocked in the presence of Triton X-100 [5]. For the common c.338C > T (p.S113 L) mutation overall lcFAO flux in fibroblasts can be normal at 37 °C culture conditions whereas at 41 °C the activity was clearly reduced in patient fibroblasts compared to controls [113]. Residual level of long-chain fatty acid oxidation in CPT2-deficient fibroblasts appears to be related to severity of disease [114, 115].

##### Molecular analysis

Around 60 mutations in the CPT2 gene (OMIM 600650) have been identified, of which most are private mutations. The c.338C > T (p.S113 L) mutation is associated with the more attenuated muscular phenotype and is relatively common with an allele frequency of up to 90% in different cohorts of patients [106, 116, 117].

### Disorders of mitochondrial long-chain fatty acid oxidation

#### Very long-chain acyl-CoA dehydrogenase (VLCAD) deficiency

##### Clinical presentation

Patients diagnosed with VLCAD deficiency (OMIM 201475) can suffer from hepatic, cardiac and muscular symptoms that can appear in infancy, but also later during adolescence or adulthood. Similar to other lcFAODs, hepatic symptoms are only seen during (early) childhood and patients with onset of symptoms at a later age predominantly experience myopathy-related complaints. Neurological symptoms, such as epilepsy and psychomotor development delay have been reported, but are usually the consequence of cerebral damage due to hypoketotic hypoglycemia. Over the past two decades, many NBS programs have included VLCAD deficiency in their panel. Before inclusion in NBS, many patients died during the first years of life often without any prior signs. One large cohort reports almost 50% deceased patients [23, 25]. Indeed, diagnosis after sudden death of infancy was more common before NBS inclusion [118, 119]. VLCAD deficiency is most frequently diagnosed of all lcFAODs worldwide with a prevalence reported between 1:31.500 to 1:94.569 [55, 120]. The majority of newly diagnosed children by NBS is asymptomatic or mildly affected because of relative high residual enzyme activity [22, 60, 121].

##### Acylcarnitine profile

Metabolites that are abnormal in VLCAD deficiency patients are usually C12, C14, C14:1, C14:2 and C16 [43], with C14:1 accumulation as most important marker, which is derived from partial breakdown of oleic acid (C18:1w9).

##### Enzymatic testing

Measurement of VLCAD activity in fibroblasts and lymphocytes can be used to confirm diagnosis and involves the conversion of palmitoyl-CoA in the presence of ferrocenium hexafluorophosphate as electron acceptor. Products (palmitenoyl-CoA and, due to enoyl-CoA hydratase activity, 3-hydroxy-palmitoyl-CoA) are separated and quantified by U(HPLC) [5, 19].

##### Molecular testing

Disease-causing mutations in the gene encoding VLCAD, i.e. *ACADVL* (OMIM 609575), are distributed all over the gene, causing a high molecular heterogeneity [122]. A follow-up analysis of 693 unrelated VLCAD-deficient patients detected by NBS underlines the molecular heterogeneity of this disorder, but also shows a relatively high prevalence of the c.848 T > C (p.V283A) pathogenic variant [51, 60].

##### Phenotype prediction

The detection of patients in the neonatal period before developing symptoms has increased the need for reliable phenotype prediction. To some extent, the phenotype correlates with genotype for VLCAD deficiency [123]. However, the continuing discovery of VUS and new mutations and frequent detection of compound heterozygosity in patients complicates phenotype prediction based on genotype in VLCAD deficiency. In a recent study in which different biochemical assays in patient fibroblasts were correlated to clinical severity, residual oleate fatty acid oxidation flux turned out to be strongly correlated with clinical severity for VLCAD deficiency. Correlation of the clinical severity with either VLCAD activity or C14/C16-acylcarnitine ratio was much less clear [124].

#### Mitochondrial trifunctional protein (MTP) deficiency/long-chain 3-Hydroxyacyl-CoA dehydrogenase (LCHAD) deficiency/long-chain 3-Ketoacyl-CoA Thiolase (LCKAT) deficiency

##### Clinical presentation

MTP is a multi-enzyme complex, consisting of three enzymes, in which different enzyme deficiencies can occur. All three enzymes are impaired in generalized MTP deficiency (OMIM 609015). Isolated LCHAD deficiency (OMIM 609016) is most common. Isolated LCKAT deficiency is extremely rare [125]. Patients with isolated LCHAD deficiency or generalized MTP deficiency share most of the clinical signs and symptoms with other lcFAODs, but are the only lcFAOD in which patients can develop peripheral neuropathy and pigmentary retinopathy [23–25, 62, 126–128]. Even though peripheral neuropathy and pigmentary retinopathy are reported in both LCHAD and MTP deficiency, retinopathy seems to be more prevalent in isolated LCHAD deficiency and peripheral neuropathy is more often seen in MTP deficiency [127–130]. There is a broad clinical spectrum, but the proportion of patients with a severe clinical presentation and who are refractory to treatment - despite early detection by NBS - is larger than seen in VLCAD deficiency [62, 131]. Some cases of hypoparathyroidism are reported for both isolated LCHAD as well as generalized MTP deficiency [132–134]. Typical for LCHAD deficiency is the increased risk for female heterozygous carriers to develop hemolysis, elevated liver enzymes, low platelets (HELLP) syndrome and acute fatty liver of pregnancy (AFLP) [135, 136]. The pathophysiology is incompletely understood, but decreased FAO of the placenta and fetus are likely to cause accumulation of long-chain 3-hydroxyacyl fatty acid metabolites in the maternal circulation. This could, in combination with increased lipase activity in the last trimester of pregnancy, have lipotoxic impact on the liver of the mother [137–139]. A meta-analysis showed an estimated clinical detection prevalence of 0.41 per 100,000 for the combination of LCHAD/MTP deficiency, this was mainly based on studies in Western populations [140]. Combined results of NBS programs in Australia, Germany and USA showed an incidence of 1:250,000 for isolated LCHAD deficiency and 1:750,000 for MTP deficiency [55].

##### Acylcarnitine profile

LCHAD, LCKAT and MTP deficiency are all characterized by accumulation of long-chain 3-hydroxyacylcarnitine species in DBS and plasma and cannot be separated from each other based on acylcarnitine profile [128]. In two separate cohort studies of LCHAD-deficient patients, the level of long-chain 3-hydroxyacylcarnitines accumulation does not increase with age, but in general, concentrations are lower after the diagnosis is known and patients are put on a long-chain fatty acid restricted diet [62, 63].

##### Enzymatic testing

Because there is no specific substrate for LCHAD, its activity is determined in the backward reaction by measuring 3-ketopalmitoyl-CoA dehydrogenase activity (LCHAD+SCHAD activity) in the presence and absence of a powerful LCHAD inhibitor (N-ethylmaleimide). The difference between the two measurements is taken to represent specific LCHAD activity [5]. When activity is reduced, mutation analysis should be performed. The thiolase activity of MTP (LCKAT) can be measured by using 3-ketopalmitoyl-CoA as substrate [5]. In case of elevated 3-hydroxyacylcarnitine levels in plasma of DBS, both the LCHAD and the LCKAT activity of MTP should be measured.

##### Molecular testing

The diagnosis is confirmed by mutation analysis of the two genes (*HADHA* (OMIM 600890) and *HADHB* (OMIM 143450)) that encode the α and ß subunits of the enzyme MTP. A mutation in one of both genes can lead to a generalized MTP deficiency due to instability of the complex [141]. In addition mutation in *HADHA* can lead to isolated LCHAD deficiency and mutations in the *HADHB* gene to isolated LCKAT deficiency. The majority of LCHAD-deficient patients carries the c.1528G > A (p.E510Q) mutation in at least one allele of the *HADHA* gene [142].

## Management of lcFAODs

### Acute management

Patients with lcFAODs are unable, or insufficiently able, to utilize fatty acids when the available glucose pool becomes depleted. Therefore, in case of metabolic crisis, emergency treatment should be aimed at supplying sufficient glucose to prevent cell damage, especially in muscles. The amount of glucose infusion depends on various factors such as residual enzyme activity, age and the level of stress due to anxiety or fever. Recommendations vary widely and there is no clear consensus.

Unfortunately, normoglycemia is not a sign that a catabolic crisis is averted. Patients may still develop rhabdomyolysis without hypoglycemia and should still receive glucose. Hyperglycemia should be treated with insulin, not with a decreasing glucose intake. Sodium and potassium should be monitored and supplemented if necessary. In case of fever, anti-pyrogenic medication should be prescribed [143]. Creatine kinase in plasma is the recommended marker to monitor rhabdomyolysis, but symptoms usually occur several hours before any rise in plasma CK levels is noticed.

### Diet and MCT

Although the understanding of the pathophysiology of lcFAODs has increased significantly in the past decades, treatment options are still limited [144]. Traditionally treatment of lcFAODs consists of prevention of catabolism, LCT restriction, and supplementation of MCT [143, 145, 146]. Intake of LCT and MCT is dependent on the specific type of lcFAOD, the residual enzyme activity, and age of the patient. In case of LCHAD/MTP deficiency and symptomatic VLCAD deficiency, MCT at 20–25% of energy intake, and total fat at 25–35% of energy intake is advised [143]. Breastfeeding is not contraindicated during LCT restriction, but should be complemented with MCT-containing formula [52, 143] . More patients have been diagnosed since the inclusion of lcFAODs in NBS panels, often before development of symptoms and it is debated whether dietary restrictions should be alleviated in certain patient groups [147, 148]. Therefore, early phenotype prediction is warranted, but this is complicated as most lcFAODs lack a clear genotype-phenotype correlation. Patients are often compound heterozygote with a combination that includes at least one novel mutations or VUS. For VLCAD deficiency, we recently proposed a dietary treatment strategy based on a functional assay that measures the overall lcFAO flux in skin fibroblasts [52]. Based on clinical and nutritional data of VLCAD-deficient patients diagnosed before introduction of VLCAD deficiency in NBS [52], LCT restriction and MCT supplementation can be alleviated under normal circumstances in patients with >10% lcFAO as long as they follow strict dietary measures during illness and a maximum feeding pause in line with the guidelines that specify maximal fasting periods per age group [143].

### Carnitine

Supplementation of carnitine is the primary therapy for OCTN2 deficiency in which very low levels of free and acylated carnitine (<10 μmol/L) in plasma occur due to abnormally high urinary loss. Complete recovery of cardiac, muscular and hepatic symptoms has been described for OCTN2-deficient patients after supplementation [149, 150]. Next to its essential role in the import of acid into the mitochondria, carnitine can also remove acyl-CoAs out of the mitochondria by facilitating their conversion to acylcarnitines which can be excreted in urine [151]. The use of carnitine in the other lcFAODs is controversial and not advised during acute management of a metabolic crisis [21, 143]. Even though supplementation can aid carnitine transport and subsequently improve FAO in theory, evidence for the efficacy of carnitine supplementation to treat secondary carnitine deficiency in lcFAODs is lacking [152]. Furthermore, concerns have been raised about the safety of carnitine supplementation in lcFAODs because it could also induce the accumulation of toxic intramitochondrial long-chain acyl-carnitines and -CoAs, and supplementation has been associated with ventricular fibrillation and rhabdomyolysis [153–155].

### Follow-up diagnostics

The frequency and choice of diagnostic follow-up for lcFAOD patients depends on the type of disorder, severity of the disease and accessibility. The monitoring of lcFAOD patients is mainly based on expert opinion (grade 4 evidence), as case-control studies are lacking [156]. In 2009, treatment recommendations have been published for lcFAODs, based on clinical data of 18 metabolic centers [21, 143]. In most centers, patients were annually seen by a metabolic specialist. For lcFAOD the specific long-chain acylcarnitines and CK levels should be measured in plasma to evaluate status of disease. Because of the risk of cardiac disease, electrocardiography and echocardiography should be performed, especially at a young age. Cardiac monitoring is usually done once every one-to-two years. This interval can be prolonged for the asymptomatic and mild patients detected by NBS, for whom the risk of cardiac disease is much smaller [22, 23, 157]. LCHAD deficiency and MTP deficiency have long-term complications not seen in other lcFAODs: progressive pigmentary retinopathy and a progressive peripheral neuropathy, which should be monitored. Furthermore anthropometric measurements should be performed during outpatient clinic visits in all lcFAOD patients, to make sure the child is thriving well, and also to detect excess weight gain at an early stage. Due to extra calories and prevention of fasting, lcFAOD patients have an extra risk to gain weight, and losing weight can be risky because this often involves fasting and reliance on lcFAO [158].

### Therapies under investigation

#### Anaplerotics

Anaplerotic therapy for lcFAOD is based on the hypothesis that a deficit in TCA cycle intermediates in these disorders hampers ATP production from substrates such as glucose, in conditions of impaired capability to produce ATP from FAO. This deficit has been shown in LCAD knockout mice, in which myocardial TCA cycle intermediates were depleted, indicating an increased but unmet need for anaplerosis [159]. Triheptanoin, a triglyceride with odd numbered fatty acids (C7), is an anaplerotic compound [160]. A recent double-blind randomized controlled trial included 32 subjects with different lcFAODs and compared the effects of a 4-month diet containing 20% of total energy from triglycerides containing either C8 or C7 fatty acids. Results seemed promising for cardiac symptoms as the left ventricle wall mass decreased and an increase in ejection fraction was observed in patients in the C7 group, but it should be noted that all the included patients had normal cardiac function [161]. Beneficial effects of triheptanoin for cardiac symptoms have been described before in a few case studies [30, 162, 163]. However, triheptanoin gave no decline in rhabdomyolysis episodes in a double-blind placebo-controlled trial [161], emphasizing the need for new therapeutic options.

#### Resveratrol and bezafibrate

Residual lcFAO can be increased in fibroblasts from VLCAD- and CPT2-deficient patients carrying missense mutations by incubation with the hypolipidemic drug bezafibrate or the polyphenol resveratrol [164, 165]. Resveratrol increases mitochondrial biogenesis through the activation of sirtuin 1 (SIRT1) and the downstream effector peroxisome proliferator-activated receptor gamma coactivator 1-alpha (PGC1α). It is likely that through this mechanism the enzyme activity can be increased if the mutated enzyme has some residual enzyme activity [166]. Bezafibrate acts as an agonist of peroxisome proliferator activator receptors (PPAR). A complete restoration of palmitate oxidation accompanied with increased protein and mRNA levels can be achieved in VLCAD-deficient fibroblasts and CPT2-deficient myoblasts of patients with considerable residual enzyme activity [167, 168]. These promising findings have led to the investigation of bezafibrate in clinical trials with CPT2-deficient patients. A clinical trial reported improved exercise tolerance and reduction of rhabdomyolysis episodes under bezafibrate treatment in all six CPT2-deficient patients tested [169]. Additionally, palmitate oxidation increased in skeletal muscle. However, another trial showed no effect of bezafibrate in CPT2-deficient patients in terms of lcFAO or exercise capacity [170, 171].

#### Ketone therapy

Ketogenic diets are often used in patients with inborn errors of metabolism, but are not suitable for lcFAOD patients, since they are unable to synthesize ketone bodies. Synthetic ketone bodies on the other hand are hypothesized to serve as an efficient alternative energy substrate that will generate sufficient acetyl-CoA for the TCA cycle to prevent symptoms. Ketone-salts have been described as therapy in multiple acyl-CoA dehydrogenase deficiency (MADD), a mitochondrial disorder that also involves impaired lcFAO, but with a more severe phenotype than lcFAODs [172]. A disadvantage of ketone salts is the undesirable salt loading, especially in patients with cardiac disease, which is why ketone-esters have more potential to serve as a new lcFAOD therapy [173, 174]. A recent study has tested the therapeutic potential of an ester of (D)-ß-hydroxybutyrate and 1,3-butanediol in VLCAD-deficient patients and found improved muscular energy balance and plasma acylcarnitine profiles after ingestion of a single dose of the ketone ester [175]. Whether daily use of this ketone ester results in alleviation of symptoms and reduces episodes of rhabdomyolysis in lcFAOD patients still needs to be investigated in a long-term follow-up study.

## Conclusions and future perspectives

With the inclusion of lcFAODs in many newborn screening programs and improvement of diagnostic techniques more patients are diagnosed but the clinical severity varies between patients. Consequently the need for prognostic markers to predict outcome is rising. In terms of treatment, up until now, dietary strategies are the best therapeutic option, but there are several other potential treatments under investigation. Nevertheless, confirmation of effectiveness of new treatments for lcFAOD is challenging, since outcome parameters to measure functional improvement are hard to find, and because it is challenging or even impossible to collect large cohorts of patients for all lcFAODs.
